# Non-invasive ventilation for acute exacerbation of COPD with very high PaCO_2_: A randomized controlled trial

**DOI:** 10.4103/0970-2113.68308

**Published:** 2010

**Authors:** Gopi C. Khilnani, Nripen Saikia, Amit Banga, Surendra K. Sharma

**Affiliations:** *Department of Medicine, All India Institute of Medical Sciences, New-Delhi, India*

**Keywords:** COPD exacerbation, non-invasive ventilation, randomized controlled trial

## Abstract

**Objective:**

To assess the role of non-invasive positive pressure ventilation (NIPPV) for management of Indian patients with acute exacerbation of chronic obstructive pulmonary disease (AECOPD).

**Materials and Methods:**

Forty patients (mean age 57.6 ± 10.8 years; M:F 31:9) with AECOPD with pH <7.35, admitted to the intensive care unit were included. Patients were randomized to receive NIPPV (N, n = 20) with conventional therapy or conventional therapy (C, n = 20) alone at admission. NIPPV was given through the nasal mask. Incidence of need of endotracheal intubation (ETI) was the primary efficacy variable. Hospital mortality, duration of hospital stay and change in clinical and blood gas parameters were the secondary outcome variables.

**Results:**

Mean pH at baseline for N and C groups were similar (7.23 ± 0.07) whereas PaCO_2_ was 85.4 ± 14.8 and 81.1 ± 11.6 mm of Hg, respectively. At one hour, patients in N group had greater improvement in pH (*P* = 0.017) as well as PaCO_2_ (P = 0.04) which corroborated with clinical improvement. Whereas need of ETI was reduced in patients who received NIPPV (3/20 vs 12/20, *P* = 0.003), in-hospital mortality was similar (3/20 and 2/20, P = NS). The mean duration of hospital stay was significantly shorter in N group (9.4 ± 4.3 days) as compared to C group (17.8 ± 2.6 days); *P* = 0.001.

**Conclusions:**

In patients with AECOPD, NIPPV leads to rapid improvement in blood gas parameters and reduces the need for ETI

## INTRODUCTION

Chronic obstructive pulmonary disease (COPD) is a major health problem and leading cause of morbidity and mortality worldwide.[1] Moreover the burden of the disease is expected to rise in future. World Health Organization has predicted that by 2020, COPD will be the 5^th^ most prevalent disease worldwide (currently ranked 12^th^) and will be among the three leading causes of death.[2] Acute exacerbations of COPD (AECOPD) are largely responsible for the morbidity and mortality associated with the disease. In fact, Andersson and colleagues have estimated that almost 35-45% of the total per capita health-care costs account for COPD exacerbations.[3]

The frequency of hypercapnic respiratory failure in patients with AECOPD varies from 16-35% with overall mortality of 35-43%.[4,5] Ventilatory support via endotracheal intubation (ETI) is the standard mode of therapy, for such patients. However, ETI is associated with several complications including nosocomial pneumonia, injury to upper airways causing ulceration, hemorrhage and long term complication like tracheal stenosis. Moreover, patients with COPD are prone to ventilator dependence and may have repeated weaning failures leading to requirement of tracheostomy.[6–9] It is obvious that avoiding ETI in patients with AECOPD is the key to improving their in-hospital outcomes. To this end, NIPPV has been claimed to be a safe and effective alternative in patients with AECOPD.

The clinical efficacy of non-invasive positive pressure ventilation (NIPPV) has been demonstrated in the management of patients with AECOPD from the West.[10–16] In comparison to medical therapy alone, NIPPV has been found to reduce the incidence of need for ETI and thereby reducing morbidity, mortality and length of hospital stay in patients with AECOPD.[13–16] However, there is relative lack of data regarding the benefit in patients with severe exacerbations. In most of the studies, the mean pH of the study group was above 7.25 and PaCO_2_ was below 80 mm of Hg.[17] Moreover, some studies excluded patients with pH<7.25.[16]

The current study was planned to determine the safety and efficacy of NIPPV in the subgroup of patients with most severe AECOPD admitted to medical intensive care unit (MICU).

## MATERIALS AND METHODS

The study was conducted at the MICU of the All India Institute of Medical Sciences, New Delhi, from March 1999 to March 2001. AIIMS is a 1600 bedded tertiary care center that caters to more than 100000 patients every year. The MICU is an 8 bedded ICU that admits patients with various critical illnesses. Due to perennial shortage of intensive care beds only the sickest patient are admitted to the MICU. Several other relatively less sick patients have to be managed at other sites.

**Figure 1:**
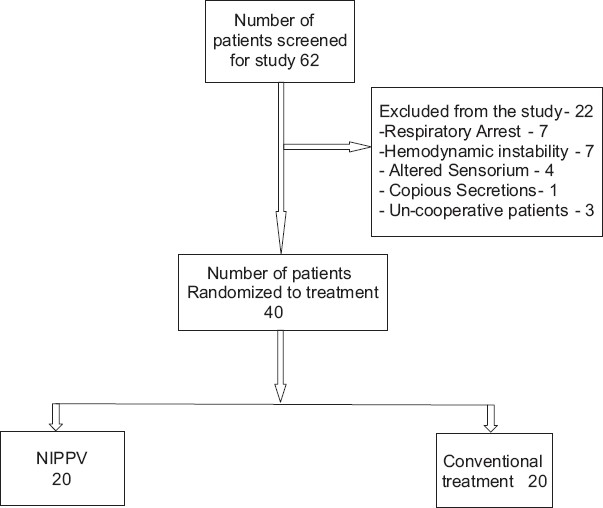
Flow chart showing patients’ enrolment

Patients with AECOPD leading to hypoxemia and respiratory acidosis with pH < 7.35 and PaCO_2_ > 45 mm of Hg admitted to the intensive care unit (ICU) were eligible for inclusion (n=62). Study was restricted to patients admitted to MICU only to ensure inclusion of patients with most severe forms of exacerbation. Diagnosis of COPD was based upon the characteristic findings on history and examination with typical radiographic abnormalities.[18] Patients’ enrolment for the study is shown in Figure 1.

Forty patients met the above mentioned criteria and were randomized to the two treatment groups. Whereas one group received conventional treatment consisting of oxygen and pharamacologic therapy (C group, n = 20), the other group were electively initiated on NIPPV in addition to the standard treatment (N group, n=20). Simple randomization using random number table was utilized for group allocation. Written informed consent was obtained from next of kin prior to enrollment. The study protocol was approved in departmental meeting which was the practice prevailed during the study period.

Baseline evaluation consisting of patient’s clinical history and detailed clinical examination was conducted. Parameters that were recorded included respiratory rate (RR), heart rate (HR), arterial blood gas analysis (ABGA) and severity of illness as assessed by APACHE (acute physiology and chronic health evaluation) II score at presentation.

### NIPPV

A bilevel positive airway pressure (BiPAP) ventilatory system (Nellcor Puritan Bennet, USA) was used for the study. This ventilator is equipped with adjustable pressure limits, and patient is ventilated as per the predefined inspiratory and expiratory airway pressure settings with each inspiration being triggered by patient’s spontaneous breath. The interface used during the study was a well fitting nasal mask (moderate or large size). After explaining the details of the process of the NIPPV institution, patient was propped up to a 45° angle. NIPPV was initiated by the investigators (GCK and NS) in all the cases. Patients were usually initiated on an inspiratory positive airway pressure (IPAP) and expiratory positive airway pressure (EPAP) of 8 cm of H_2_O and 4 cm of H_2_O respectively. Subsequent adjustments were carried out according to the need of the patient and the results of blood gas analysis. The protocol was to augment IPAP and EPAP by 2 cm H_2_O every 5-10 min, patient’s comfort and arterial oxygen saturation permitting. All patients were given oxygen at 3-4 l/min during ventilation to maintain oxygen saturation above 90%.

Each patient was encouraged to use the NIPPV up to 16 hrs/day including day and night and duration of ventilation was recorded in each patient accordingly. NIPPV was discontinued for eating and drinking.

After starting treatment each patient was monitored closely for initial one hour. patient’s discomfort and intolerance to mask was looked for. Clinical status such as use of accessory muscles of respiration, increase or decrease of dyspnoea, appearance or disappearance of cyanosis, heart rate, respiratory rate and blood pressure were monitored. Level of consciousness was also closely monitored.

Patient assigned to C group received oxygen at a rate of 3-4 L/ min by means of nasal prongs or oronasal mask. Both groups received pharmacologic treatment including bronchodilators [inhaled salbutamol, ipratropium bromide, subcutaneous terbutaline, and steroids (IV hydrocortisone)] as per the standard guidelines.[19] Intravenous (IV) antibiotics were given to all patients at admission and subsequently the duration of antibiotic was decided on case to case basis by the ICU treating team.

Continuous arterial oxygen saturation was monitored using pulse oxymeter. ABGA was done at 1 hr, 6 hr, 24 hr, 48 hr, 72 hr, on 5^th^ day and any other time if patient’s condition required so. Patients who deteriorated in terms of gas exchange parameters (rising PaCO_2_ and/or worsening pH), level of consciousness (Glasgow coma scale < 8), or haemodynamic stability (mean arterial pressure < 60 mm of Hg) as well as those with copious secretions and inability to tolerate face mask from either group were intubated and initiated on conventional mechanical ventilation.

Presence of sustained clinical improvement with reduction of RR <24/ min, HR <100/ min and presence of normal pH, PaCO_2_ < 55 mm of Hg and O_2_ saturation >90% on ABGA were required before patients were considered for weaning from NIPPV in the N group.

Incidence of need of ETI was the primary outcome variable. Hospital mortality, duration of hospital stay and change in clinical and blood gas parameters were the secondary outcome variables. Various complications including adverse effects related to the procedure of NIPPV such as aspiration, bloating and skin ulcers, development of ventilator associated pneumonia and hemodynamic instability in the two groups were recorded and compared between the two groups as safety variables.

### Statistical analysis

Categorical variable were described in proportions whereas continuous variable were described using mean ± standard deviation. Comparisons were made between the baseline data and post admission data within the two groups as well as between the two groups. Whereas continuous variable were compared between two groups using independent t- test, paired t- test was used for intra-group comparisons. Multiple comparisons were performed using repeated measures of analysis of variance (ANOVA). Chi square test was used for comparisons between categorical variables. Significance was considered at *P* <0.05 (two tailed).

## RESULTS

The baseline characteristics of the two groups were similar and are shown in Table 1. In the N group, the mean IPAP used was 15.5 ± 3.4 cm of H_2_O and EPAP was 9.9±1.9 cm of H_2_O. Maximum IPAP used was 18 cm of H_2_O and EPAP was 11 cm of H_2_O.

**Table 1:** Baseline characteristics of the two treatment groups

Characteristic of patients	N Group	C group	*P* value
Age (years)	55.25 ± 10.09	60 ± 11.07	NS
Male/female	15/5	16/4	NS
Smoker	17 (85)	16 (80)	NS
Chronic cor pulmonale	6 (30)	9 (45)	NS
Pneumonia at admission	8 (40)	9 (45)	NS
Old treated pulmonary tuberculosis with pulmonary fibrosis	3 (15)	4 (20)	NS
Bronchiectasis	2 (10)	0	NS
Essential hypertension	2 (10)	2 (10)	NS
Hypothyroidism	1 (5)	1 (5)	NS
Pneumothorax	1 (5)	0	NS
APACHE II	15.70 ± 2.32	17.17 ± 4.83	NS
Heart rate	120.3 ± 15.5	111.6 ± 13.30	NS
Respiratory rate	34.85 ± 4.738	35.31 ± 4.831	NS
pH	7.229 ± 0.074	7.228 ± 0.073	NS
PaCO_2_ (mmHg)	85.370 ± 14.85	81.09 ± 11.65	NS
PaO_2_ (mmHg)	61.18 ± 14.7	61.50 ± 15.06	NS
HCO_3_^-^ mEq/l	35.40 ± 6.16	35.65 ± 4.69	NS
O_2_ saturation (%)	88.26 ± 4.96	90.61 ± 5.67	NS

N group: NIPPV group; C group: Conventional therapy group; NS: not significant; APACHE II: Acute Physiology and Chronic Health Evaluation II, Figures in parentheses are in percentage

### Primary outcome variable

The need of ETI was reduced significantly by use of NIPPV. Twelve patients out of 20 (60%) in C group required ETI as compared to 3 out of 20 (15%) in N group (*P* = 0.003). Rise in PaCO_2_ with or without worsening in the level of sensorium was the indication for ETI in most of the patients (both patients in N group and 10 out of 12 patients in C group).

### Secondary outcome variables

The most striking effects of the institution of NIPPV on the blood gas parameters were noticed in the first hour of initiation itself. In the N group, a statistically significant change was noted within one hour in both pH and PaCO_2_. Whereas the mean pH rose from a baseline of 7.23 ± 0.07 to 7.27 ± 0.08 (*P* <0.001), mean PaCO_2_ fell from a baseline value of 85.4 ± 14.8 mm of Hg to 65.1 ± 37.6 mm of Hg (*P* = 0.04). In contrast, there was no significant change in the mean pH (7.23 ± 0.07 to 7.22 ± 0.08) and PaCO_2_ (81.1 ± 11.7 mm of Hg to 86.2 ± 20.6 mm of Hg) levels during this period in the C group. Consequently, the difference in mean pH and PaCO_2_ levels between the two groups at one hour had become statistically significant (that were similar at the baseline, see Table 2). There was no significant difference in the change in the other parameters between the two groups [Table 2]. Subsequently, by the end of 6 hours, the difference in the mean pH and PaCO_2_ levels between the two groups was lost and the pH and PaCO_2_ levels of the C group tended to approximate those of N group [Table 3]. So was the case in all subsequent recordings [Table 3].

**Table 2:** Change in clinical and blood gas parameters within the first hour of initiation of treatment in both the groups

	0 hr (n = 20)	*P* value	1 hr (n = 20)	*P* value
Heart rate	120.31 ± 15.96 (N) 112.42 ± 13.36 (C)	NS	119.05 ± 14.32 (N) 114.94 ± 12.84 (C)	NS
Respiratory rate	34.85 ± 4.73 (N) 35.31 ± 4.83 (C)	NS	33.95 ± 5.65 (N) 35.89 ± 5.55 (C)	NS
pH	7.228 ± 0.07 (N) 7.229 ± 0.074 (C)	NS	7.274 ± 0.08 (N) 7.217 ± 0.086 (C)	0.017
PaCO_2_ (mm Hg)	85.37 ± 14.85 (N) 81.09 ± 11.65 (C)	NS	65.13 ± 37.63 (N) 86.20 ± 20.58 (C)	0.024
PaO_2_ (mm Hg)	61.18 ± 14.73 (N) 61.50 ± 15.06 (C)	NS	67.4 ± 20.09 (N) 64.10 ± 26.07 (C)	NS
HCO_3_^-^ mEq/l	35.40 ± 6.16 (N) 35.65 ± 4.69 (C)	NS	35.47 ± 7.53 (N) 36.67 ± 5.78 (C)	NS
O_2_ saturation (%)	88.78 ± 4.96 (N) 90.05 ± 6.02 (C)	NS	90.78 ± 5.57 (N) 92.00 ± 5.94 (C)	NS

NS: not significant; Letters in parentheses (C and N) indicate the treatment group

**Table 3:** Serial arterial pH and PaCO_2_ values in the two treatment groups

Time	pH (Mean±SD)	*P* value	Mean	*P* value
0 hr	7.23±0.07 (N) (n=20) 7.23±0.07 (C) (n=20)	NS	85.4±14.8 (N) (n=20) 81.1±11.6 (C) (n=20)	NS
1 hr	7.27±0.08 (N) (n=20) 7.22±0.09 (C) (n=20)	0.017	65.1±37.6 (N) (n=20) 86.2±20.6 (C) (n=20)	0.024
6 hr	7.32±0.09 (N) (n=19) 7.29±0.09 (C) (n=13)	NS	67.2±13.5 (N) (n=19) 67.3±13.9(C) (n=13)	NS
24 hr	7.37±0.08 (N) (n=18) 7.35±0.1 (C) (n=10)	NS	58.1±24.3 (N) (n=18) 68.4±18.5(C) (n=10)	NS
48 hr	7.36±0.1 (N) (n=18) 7.38±0.11 (C) (n=10)	NS	56.9±16 (N) (n=18) 62.8±17.5 (C) (n=10)	NS
72 hr	7.39±0.08 (N) (n=18) 7.39±0.05 (C) (n=8)	NS	62.2±13.4 (N) (n=18) 62.2±13.5 (C) (n=8)	NS
5^th^ day	7.39±0.09 (N) (n=17) 7.40±0.05 (C) (n=8)	NS	59.7±8.4 (N) (n=17) 59.5±8.6 (C) (n=8)	NS

SD: standard deviation; NS: not significant; Letters in parentheses (C and N) indicate the treatment group and the respective number of patients in each group

Out of the 20 patients in the N group, 4 (20%) developed complications during NIPPV. One patient developed aspiration pneumonia, two patients complained of abdominal bloating sensation and irritation in eyes and one patient had upper GI bleed. No patient developed skin ulcers on NIPPV. On the other hand, half of the patients in C group (n = 10) developed some complication namely nosocomial pneumonia (n = 4), upper GI bleed (n = 3), pneumothorax (n = 2) and paroxysmal supraventicular tachycardia (n = 1).

The mean duration of hospital stay in the N group was 9.4 ± 4.3 days as compared to 17.8 ± 2.6 days in the C group (*P* = 0.001). There was no significant difference in the hospital mortality of the two groups (3/20 in the N group versus 2/20 in the C group, *P* = NS). The cause of death in the N group were septicemia (n = 2) and acute coronary event (n = 1) and septicemia in the C group (n = 2).

## DISCUSSION

The present study demonstrated that NIPPV is an effective and safe modality for initial management of patients with severe exacerbation of COPD. Earlier studies from the West have demonstrated the utility of this modality in patients with COPD[10–14] and the current work validates the findings in Indian patients. Moreover it also confirms the benefit of NIPPV in patients with much more severe form of exacerbation. Previous studies from India have reported the use of NIPPV in patients with acute respiratory failure (ARF) due to varied etiologies.[20–22] Singh *et al*, showed that NIPPV use was associated with significant improvement in clinical and ABG parameters in patients with ARF.[20] Later, George *et al*., demonstrated benefits of NIPPV in avoiding the need for invasive mechanical ventilation in patients presenting with ARF of diverse etiology.[21] The largest published trial which included 248 patients with ARF, compared the role of NIPPV in patients with ARF due to COPD and other diseases. It concluded that NIPPV is more effective in preventing endotracheal intubation in ARF due to COPD than other causes.[22] Notably, these studies have included patient with ARF of varied etiology, therefore, the cohorts are heterogenous as compared to our study which describes the use of NIPPV in COPD patients only.

The current study comprised of patients significantly different from those included in the earlier studies. In the spectrum of severity of the exacerbation, those with the most severe form of exacerbation as reflected by mean pH and mean PaCO_2_ were studied. Majority of patients had severe acidemia (pH<7.25: n = 23, 57.5%; pH<7.30: n = 31, 77.5%) and hypercapnia (PaCO_2_>80 mm of Hg: n = 26, 65.0%; PaCO_2_>75 mm of Hg: n = 31, 77.5%).

The study group comprised of a typical COPD population with majority patients being males, smokers and above the age of 50 years. Both the groups were evenly matched in terms of demographics, co-morbid conditions, severity of illness and baseline clinical and blood gas parameters.

Whereas the study was not powered to demonstrate mortality benefit, need of ETI was reduced via use of NIPPV. Moreover, the duration of hospital stay as well as complications during hospitalization were also reduced. All these findings reflect the significant morbidity advantage that NIPPV use provides in patients with acute exacerbation of COPD. Given a larger sample size, these advantages are likely to convert into a mortality benefit as has been demonstrated in some of the earlier studies from outside India[12,13] although this may not be the case always.[23] The present study also showed that NIPPV via nasal mask is well tolerated in patients with acute respiratory failure secondary to COPD and it can be used safely with only a few complications. NIPPV had to be discontinued due to intolerance to mask in only one patient and no other major complication such as ulcerations were noted in the N group.

Whereas the reduction in the need of ETI and the consequent complications including the increased hospital stay are the obvious effects of NIPPV, it is interesting to examine the effects of this therapeutic modality on other parameters. It was documented that NIPPV institution had absolutely no impact on the clinical parameters such as heart rate and respiratory rate. Moreover, the level of oxygenation was also not affected. However, the major departure from the usual pattern (that was seen in the C group) was in terms of early and sustained reduction in the PaCO_2_ levels and the consequent rise in pH. Within the first hour of initiation of NIPPV itself, there was a marked improvement in arterial pH and PaCO_2_ levels. The improvement was statistically significant both in terms of intra-group variations (in comparison to baseline) as well as inter-group in terms of being significantly lower than the mean levels in the C group. Whereas by the end of 6 hours both pH and PaCO_2_ levels tended to approximate and the difference was lost, it must be interpreted in light of the fact that these mean values were of a sub-group of patients with in the C group (n = 13). The other seven patients had already met the end point of ETI (more than half of the total patients who eventually required ETI from C group) and their data were not included in these values. On the other hand a large majority of patients in the N group (n = 19) did not meet the endpoint of ETI by the end of 6 hours. These are pertinent findings and bring out a couple of practical points. One, patients with exacerbation of COPD tend to worsen very fast and a majority of these patients would end up requiring ETI within the initial few hours itself. It seems that these patients are already on the sharp downhill slope of the respiratory load and capacity mismatch and therefore the ‘window period’ between the time when these patients present to the hospital and end up needing ETI is small. Unless, a significant intervention in terms of either reducing the respiratory load (that is achieved to variable extents with institution of bronchodilator therapy) or improving the respiratory capacity (that tends to go unattended in conventionally managed patients but is addressed by institution of NIPPV) is initiated, these patients would continue to deteriorate and end up needing ETI. Secondly, as reflected by the rapid improvements in the blood gas parameters, it is obvious that NIPPV starts to work immediately. There is an obvious immediate and effective unloading of respiratory muscles and reduction in the work of breathing, the impact of which is an immediate improvement in blood gas parameters. These improvements are a reflection of the bridging of the gap between respiratory load and capacity and eventually convert into reduction in the incidence of need of ETI. Moreover, it is safe to deduce that the initial response in terms of blood gas parameters, especially pH and PaCO_2_, can be taken as predictors of success of NIPPV in COPD patients. If these parameters worsen or remain unchanged after one hour of institution of NIPPV, the patient must be monitored very closely for need of ETI.

There are certain shortcomings of the present work that need to be mentioned. This was a relatively small study and, at best, only adds to the weight of the existing evidence. Moreover, because of the small sample size, subgroup analysis to determine the correlates of success or failure of NIPPV in COPD patients could not be determined. The current study evaluated the NIPPV in AECOPD patients managed in intensive care set up where state of the art monitoring along with trained medical staff was available. Nonetheless, Plants and co-workers has also reported the use of NIPPV as safe and effective in appropriately selected patients managed in the respiratory wards.[16] Another limitation of our study is the use of nasal mask instead of the full face mask. The nasal mask although is, usually, associated with better compliance, however, ventilatory support may be compromised due to pressure leak. However, prior to this study we were using nasal mask routinely in patients with COPD. The results of our study have shown that NIPPV using nasal mask may also be an effective way of ventilatory support in these patients.

It is concluded that NIPPV is a promising therapeutic modality for management of patients with exacerbations of COPD. Its timely institution leads to a rapid and profound improvement in blood gas variables that culminates into reduction in the incidence of need of ETI in these patients.
